# Causes of short stature in Pakistani children found at an Endocrine Center

**DOI:** 10.12669/pjms.326.11077

**Published:** 2016

**Authors:** Ali Jawa, Syed Hunain Riaz, Muhammad Zaman Khan Assir, Bahjat Afreen, Amna Riaz, Javed Akram

**Affiliations:** 1 Prof. Ali Jawa, MD, MPH, DABIM, FACE, Wilshire Cardiovascular and Endocrine Center of Excellence (WILCARE), Lahore, Pakistan, Shaheed Zulfiqar Ali Bhutto Medical University, PIMS, Islamabad, Pakistan; 2 Syed Hunain Riaz, FCPS (MED), Wilshire Cardiovascular and Endocrine Center of Excellence (WILCARE), Lahore, Pakistan; 3 Muhammad Zaman Khan Assir, FCPS (MED), Wilshire Cardiovascular and Endocrine Center of Excellence (WILCARE), Lahore, Pakistan; 4 Bahjat Afreen, FCPS (MED), Wilshire Cardiovascular and Endocrine Center of Excellence (WILCARE), Lahore, Pakistan; 5 Amna Riaz, FCPS (MED), FCPS (ENDO), Wilshire Cardiovascular and Endocrine Center of Excellence (WILCARE), Lahore, Pakistan; 6 Prof. Javed Akram, MD, MRCP, FRCP (GLASG), FACP, FACC, FASIM, Shaheed Zulfiqar Ali Bhutto Medical University, PIMS, Islamabad, Pakistan

**Keywords:** Children, Growth hormone deficiency, Hypothyroidism, Pakistani, Short stature, Vitamin D deficiency

## Abstract

**Background and Objective:**

Short stature is defined as height below 3^rd^ centile. Causes of short stature can range from familial, endocrine disorders, chronic diseases to chromosomal disorders. Most common cause in literature being idiopathic short stature. Early detection and management of remedial disorders like malnutrition and vitamin D deficiency, Endocrine disorders like growth hormone deficiency & hypothyroidism can lead to attainment of expected height. Pakistani data shows idiopathic short stature as the most common cause of short stature. Our study aimed at detecting causes of short stature in children/adolescents at an Endocrine referral center.

**Methods:**

A retrospective study was conducted at WILCARE Center for Diabetes, Endocrinology & Metabolism, Lahore on 70 well-nourished children/adolescents. The patients had been evaluated clinically, biochemically and radiologically as needed. Biochemical testing included hormonal testing as well to detect endocrine causes. Data was entered and analyzed in SPSS 20.0.

**Results:**

Leading cause of short stature in our population was Growth Hormone (GH) deficiency seen in 48 out of 70 (69%) patients. Second most common endocrine abnormality seen in these patients was Vitamin D deficiency [44 out of 70 patients (63%)]. Primary hypothyroidism; pan-hypopituitarism & adrenal insufficiency were other endocrine causes. The weight for age was below 3^rd^ percentile in 57 (81%) patients, with no association with other major causes.

**Conclusion:**

Growth hormone and Vitamin D deficiency constitute one of the major causes of short stature among well-nourished children with short stature in Pakistan.

## INTRODUCTION

Short stature is defined as height less than or equal to 2 SD below the mean for the age and sex or height below 3^rd^ centile.^1^ Causes of short stature can either be normal variants of growth like familial short stature and constitutional delay of growth and puberty(CDGP)^2^,^3^ or it can be a result of chronic systemic diseases (renal, pulmonary and cardiac diseases, coeliac disease)^4^,^5^ endocrine disorders (hypopituitarism, isolated growth hormone deficiency, isolated hypothyroidism, endogenous & exogenous Cushing syndrome, adrenal insufficiency, osteomalacia or rickets),^6^-^9^ genetic (cystic fibrosis),^10^ chromosomal disorders (Turners syndrome)^11^ and skeletal dysplasias.^12^ Malnutrition, chemotherapy, radiotherapy^13^ and surgery are also contributory to short stature. The most common causes of short stature reported in literature are CDGP and familial short stature^14^-^17^ collectively called ‘Idiopathic short stature’ and is primarily a diagnosis of exclusion.

Final adult height is determined by a child’s genetics. In familial short stature the child reaches within the height range of the parents/family.^18^ In CDGP there are subtle changes within the pituitary-gonad axis as well as the growth hormone-IGF-1 axis which results in delayed skeletal maturation and puberty spurt. This results in height shorter than the predicted adult height.^19^ However it is seen that children with CDGP also have familial short stature in up to 40% of patients.^20^ Remediable causes like coelic disease or malnutrition when detected early can allow for attainment of normal projected height. Endocrine causes like idiopathic growth hormone deficiency and hypothyroidism when detected early and treated can lead to height within normal range.^21^,^22^ Pakistan is a developing country and nutritional deficiency is prevalent in the children.^23^ 33% of children under 5 years of age are underweight, while 53% are stunted.^24^ Vitamin D deficiency is a present in up to 94% of our children, which can reduce skeletal mineralization and bone growth rate.^25^ Data from Pakistan on etiology of short stature in children is sparse however existing data shows Constitutional delay and Familial short stature (normal variants of growth) as the major causes.^14^-^16^ The current local data is from tertiary health care institutions however our study aims at detecting causes of short stature in children/adolescents presenting to WILCARE Center for Diabetes, Endocrinology & Metabolism, Lahore which is a Endocrine referral center. In our study we expected to find a more broader range of endocrine causes with emphasis on growth hormone deficiency.

## METHODS

The study was conducted at WILCARE Center for Diabetes, Endocrinology & Metabolism, Lahore. WILCARE center is a referral center for endocrine and metabolic disorders. Data for seventy (70) children/adolescents who presented with short stature to WILCARE Center for Diabetes, Endocrinology & Metabolism, Lahore was extracted from the electronic database and retrospective descriptive study was conducted. Consent for use of the data for this study was taken telephonically from the parents/guardians of the children/adolescents. Patients were included who had been labeled as having short stature based on height less than -2 SD or less than 3rd centile for age and gender plotted on 2000 CDC growth charts (developed by the American National Center for Health Statistics in collaboration with the National Center for Chronic Disease Prevention and Health Promotion).^26^ The patients mostly belonged to middle to upper socioeconomic strata of the society. These patients had been evaluated clinically, biochemically and with imaging as was needed. Clinical evaluation had been done by taking a detailed history (including antenatal, perinatal, postnatal history) and performing thorough physical examination. Based on clinical evaluation, relevant biochemical profile which included complete blood count, urine and stool examination and blood glucose, blood urea, serum creatinine, liver function tests, electrolytes, fasting serum calcium, phosphorus and alkaline phosphatase and urinary pH were obtained. Hormonal testing including Serum TSH and free T4 assay, IGF-1 levels, 8 am serum cortisol, and vitamin D levels were performed on Architect instrument employing chemiluminescent microparticle immunoassay (CMIA). Growth Hormone deficiency had been diagnosed with serum IGF-1 levels less than the normal range for age and sex. Hypothyroidism had been diagnosed on basis of TSH and/or FT4 values. Additional tests like Insulin tolerance test for Growth hormone deficiency, anti-Tissue Transglutaminase antibodies, iron studies, FSH and karyotyping had been performed in selected cases and MRI pituitary with and without contrast performed when biochemical evidence of pituitary hormone disorders was evident.

In all cases, X-ray hand was done to assess the bone age using Greulich and Pyle’s Standards.^27^ The collected data was entered and analyzed in SPSS version 20. Descriptive statistics were used to calculate frequencies of the various causes of short stature. Categorical variables were compared using Chi-square and Fisher exact tests. A p value < 0.05 was considered to be statistically significant.

### Ethical approval

All procedures performed in studies involving human participants were in accordance with the ethical standards of the institutional and/or national research committee and with the 1964 Helsinki declaration and its later amendments or comparable ethical standards.

## RESULTS

A total of 70 patients with short stature were included in the analysis. The median age at the time of evaluation was 11 years (range 2 to 18 years) and 38 (54%) were male. The weight for age was below 3^rd^ percentile in 57 (81%) patients.

The leading cause of short stature in our population was Growth Hormone (GH) deficiency seen in 48 out of 70 (69%) patients.(Table-I) Second most common endocrine abnormality seen in these patients was Vitamin D deficiency [44 out of 70 patients (63%)]. Isolated Vitamin D deficiency was seen in only 4 (6%) cases; it was most commonly seen in combination with other endocrine abnormalities. GH deficiency was associated with Vitamin D deficiency, though the association was insignificant. Primary hypothyroidism was found in 6 (9%) patients. Panhypopituitarism was the cause of short stature in 4 (6%) cases. Growth hormone deficiency and Vitamin D deficiency were equally distributed across both genders.

**Table-I:** Causes of Short Stature.

Condition	Number of patients (Total N = 70)	Percentage
GH deficiency	48	69
Vitamin D deficiency*	44	63
Primary hypothyroidism	06	09
Panhypopituitarism	04	06
IUGR	03	04
Steroid related stunting of growth	03	04
Iron deficiency anemia	02	03
Turner Syndrome	01	01
Premature ovarian failure	01	01
Achondroplasia	01	01
Primary adrenal insufficiency	01	01
Central adrenal insufficiency	01	01
Central hypothyroidism	01	01
Lipid storage disease	01	01

* Vitamin D deficiency was seen in most patients along with other endocrine abnormalities, therefore the sum of all these conditions does not correspond to total number of patients.

History of intrauterine growth retardation (IUGR) was documented in 3 (4%) patients. three (4%) patients had history of asthma and steroid associated stunting of growth. Iron deficiency anemia as evidenced by microcytic anemia, low serum ferritin and high TIBC was seen in 2 (3%) patients. Rare causes of short stature seen in one patient each (1%) included Turner Syndrome, premature ovarian failure, achondroplasia, primary adrenal insufficiency, central adrenal insufficiency, central hypothyroidism and lipid storage disease. Weight below 3^rd^ centile was cross tabulated against Growth hormone deficiency, Primary Hypothyroidism, Pan-hypopituitarism and Vitamin D deficiency and no significant association was found.

## DISCUSSION

About 3% of children have short stature and further evaluation is warranted in these patients. There is abundance of research on short stature in children/adolescents globally. In contrast there are far fewer studies conducted in Pakistan. Growth Hormone (GH) deficiency was by far the most common cause of short stature in our study, in 48 out of 70 patients (69%). This is a trend different from what has been reported in studies globally and other studies regionally where normal variants of growth (constitutional delay of growth and puberty and familial short stature) have been reported as predominant cause either separately or in combination. Knoop et al. from Germany reported that 68% cases of short stature were of CDGP or familial short stature.^9^ Other studies have also reported similar results where normal variants of growth constituted 52-85% of cases of short stature.^16^,^28^-^30^ The likely explanation of this disparity is that firstly, these studies were population based compared to our specialist referral center based study and secondly, in most of the studies patients were recruited in tertiary health care centers with patients mostly from lower socio-economic strata. In contrast, our study included patients mostly from upper/middle socioeconomic status.

Prior studies have shown that GH deficiency is the second leading cause of short stature. A prevalence of 8% to 23% has been reported in various studies on short stature.^28^,^29^,^31^ Vitamin D has an established role in bone health and growth in children^32^ and its deficiency has been reported world over.^33^,^34^ In Pakistani population, vitamin D deficiency has been reported in up to 94% of individuals.^25^ In our study, the second most common endocrine abnormality seen in patients with short stature was Vitamin D deficiency (63%). Vitamin D deficiency was found occurring mostly with other endocrine causes of short stature and isolated deficiency was seen in only 6% of cases (n=4). This possibly is a reflection of the Vitamin D deficiency in general Pakistani population.^35^

Thyroid hormone has an important role in bone development and linear growth in children and deficiency of thyroid hormone can lead to stunted growth and/or maturation arrest.^36^ Primary hypothyroidism was seen in 9% (n=6) cases, while central hypothyroidism was present in only 1% in our study. Sultan et al. in a Pakistani study reported hypothyroidism as a cause of short stature in 5.6% of their study population,^14^ while another local study by Rabbani et al. reported it as 17%.^15^ Pan-hypopituitarism which is known to stunt growth was present in 6% of the patients, an Iranian study reported it as 3.5%.^31^ The weight below 3^rd^ centile in 81% of our study population is of special interest as no etiological factors were identified and especially when our children/adolescent population belonged to the upper/middle socio-economic class. Low weight for age has not been specifically elaborated in previous studies, in which its utility has been to categorize patients into disorders of malabsorption. Further research is required to identify possible causative factors.

### Limitations of the Study

This study was conducted at an Endocrine Clinical referral center and may not actually represent the true frequency of causes of short stature in our population. Large scale population based studies will give a better estimate of causes of short stature.

## CONCLUSION

Growth hormone and Vitamin D deficiency constitute one of the major causes of short stature among well-nourished children with short stature in Pakistan. Early recognition of these pathologies can not only help attain normal stature but also improve their quality of life and social standing. General Physicians need to be cognizant of and refer patients promptly to endocrinologist for timely treatment.
